# Informal care after hip fracture: prospective cohort

**DOI:** 10.1186/s12877-024-05040-y

**Published:** 2024-05-17

**Authors:** Jonas Ammundsen Ipsen, Bjarke Viberg, Lars T. Pedersen, Eva Draborg, Inge Hansen Bruun

**Affiliations:** 1 Department of Physical Therapy and Occupational Therapy, Lillebaelt Hospital, University Hospital of Southern Denmark, Kolding, Denmark; 2 Department of Regional Health Research, University of Southern Denmark, Odense, Denmark; 3 Department of Orthopaedic Surgery and Traumatology, Lillebaelt Hospital, University Hospital of Southern Denmark, Kolding, Denmark; 4 Department of Orthopaedic Surgery and Traumatology, Odense University Hospital, Odense, Denmark; 5 Department of Clinical Research, University of Southern Denmark, Odense, Denmark; 6 Department of Health Education, University College South Denmark, Esbjerg, Denmark; 7 Department of Public Health, DaCHE - Danish Centre for Health Economics, University of Southern Denmark, Odense, Denmark

**Keywords:** Informal care, Hip fracture, Care, Prospective

## Abstract

**Background:**

Hip fracture is very common and it has life-shattering consequences for older persons. After discharge the older persons need help with even basic everyday activities from formal and informal caregivers. In Scandinavia formal care are well-developed however the presence of informal caregivers likely reflect on the amount of formal care and wears on the informal caregivers. This study explore how often and how much informal care (IC) older persons receive after hip fracture.

**Method:**

We contacted 244 community-dwelling older persons every two weeks the first twelve weeks after discharge after hip fracture and asked them if they received care from family and/or friends and how much. We used non-parametric statistics and level of significance was 95%.

**Results:**

The proportion of older persons receiving IC was 90% and the median amount of IC was 32 hours (IQR 14-66). The number of older persons who received IC was highest the first four weeks after discharge and so was the amount of hours of IC. The older persons that were high-dependence on IC received a median of 66 (IQR 46-107) hours compared to the low-dependent of 11 hours (IQR 2-20).

**Conclusion:**

IC is very frequent, especially the first two to four weeks after discharge. The median IC was 32 hours from discharge to the 12-week follow-up. However, this figure tended to rise for persons with, among other, reduced functionality and those residing with a partner.

**Implications:**

With respect to local differences, the findings in this study are likely applicable to other Scandinavian countries. We strongly suggest that the variation in older person need for informal caregiver be given consideration in the prioritisation of resources.

**Trial registration:**

This prospective cohort study of informal care, was part of a cluster-randomised stepped-wedge clinical controlled trial. Written consent was obtained required by regional ethics committee S-20200070. Data was collected in accordance with the Danish Data Protection Agency (20-21854).

**Supplementary Information:**

The online version contains supplementary material available at 10.1186/s12877-024-05040-y.

## Background

Hip fracture is the most common surgically treated trauma and it has life-shattering consequences for older persons [1, 2]. Upon discharge to home, older persons face challenges with basic activities such as walking or getting dressed, incurring an increased need for assistance [1, 2]. To meet this need, older persons receive formal care from healthcare professionals and/or informal care (IC) from family or friends [3–6].

The United Nations Economic Commission for Europe Standing Working Group on Ageing warns that without adequate support the negative influence on the physical and mental health of IC providers can increase demands and costs of health care [7]. Compared with other member countries of the United Nations, Scandinavian countries have a universal healthcare system in which the public is obliged to provide care and family and friends are not bound to provide IC [7, 8]. However, in contrast, the Scandinavian countries have the highest prevalence of informal caregivers in Europe [9]. Thus, informal caregivers likely want to take care of their older relatives despite the duty of the public health care system. This, in combination with an increased focus on resource scarcity, can have increased the healthcare system’s dependency on informal caregivers when frail older persons are discharged to their own homes after hip fractures [10, 11].

In Sweden, Finland and Denmark, 13-16% of the population are informal caregivers, and Danish and Norwegian older persons with high needs for formal care also receive significant amounts of IC [11–14]. Denmark, Sweden, Norway and Finland all have a high prevalence of IC, and in all four countries, there are recommendations on the inclusion of informal caregivers in meeting patients’ need for help [11, 14–18]. There are likely differences in how these recommendations are employed between countries. Nevertheless, all four countries have a healthcare system divided in sectors with partly autonomous municipalities and hospitals. Thus, healthcare professionals, patients and informal caregivers across Scandinavia likely face similar challenges to coherent care when discharging patients after hip fractures.

Although IC is probably common among older persons after hip fracture in Denmark, the frequency and amount of this IC have not been assessed before in a Scandinavian country. Filling this knowledge gap is important as it provides insight into the burden of IC on family and friends after hip fracture. Thus, this study aimed to quantify the frequency and amount of IC received by home-dwelling persons aged 65 and older after hip fracture.

## Methods

### Study design

This study, a prospective cohort study of informal care, was part of a cluster-randomised stepped-wedge clinical controlled trial (‘Rehabilitation for Life’) [19]. Reporting followed the guidelines for Strengthening the Reporting of Observational Studies in Epidemiology (STROBE).

### Setting

The cohort encompassed one catchment area (one hospital and six municipalities serving a mixed rural and urban population). The responsibility for providing care, which is offered free of charge, is shared between hospital and municipalities. Municipalities regularly assess whether the amount of care is sufficient or requires an increase or decrease with regard to the older person’s needs; this can ultimately become a lifelong service [20, 21].

### Participants

Inclusion criteria were community-dwelling persons aged 65 years or older after hip fracture treated at a one hospital in Southern Denmark. Exclusion criteria were inability to speak or understand Danish, discharge to permanent residence in nursing homes, progressed dementia, and refusal to participate in the trial, refused to participate in this study or having short life expectancy.

### Outcomes

The primary outcome was the number and percentage of older persons receiving IC from time of discharge to follow-up at 12 weeks.

The secondary outcome was the median total number of hours of IC from discharge to 12-week follow-up.

The biweekly change in frequency and number of hours of IC was explored with and without inclusion of the older persons with missing information.

### Variables

*Informal care:* the proportion of older persons receiving assistance from informal caregivers from time of discharge to 12-week follow-up.

*Amount of informal care:* the aggregated number of hours of IC the older persons received from informal caregivers from time of discharge to 12-week follow-up.

*Biweekly change in frequency and amount of IC:* the number of older persons receiving IC and the median number of hours of IC in weeks 1-2, weeks 3-4, weeks 5-6, weeks 7-8, weeks 9-10 and weeks 11-12.

*Demographic characteristics:* age, gender, body mass index (BMI), living arrangement (i.e., alone, cohabiting or other), and physical status classification using anesthesiologist’s pre-surgery validation American Society of Anaesthesiologists (ASA) levels one being the best. The ASA score assess patient’s overall health based on five classes [22]. In this study, the ASA score was dichotomised as ≤2 or above 2.

*Type of operation:* categorised as arthroplasty, sliding hip screw or intramedullary nail.

*Mobility:* New Mobility Score (NMS) was a clinician-applied 0-9 score measured at discharge. A higher score indicates better mobility [23].

*Basic mobility:* Cumulated ambulation score (CAS) was a clinician-applied 0-6 score measured at discharge. Higher score indicated better basic mobilisation [24].

*Activities of daily living:* Barthel-20 was measured on a scale from 0-20, at discharge, to assess a patient’s need for assistance. Higher score indicate lesser need for help [25].

*Overall health:* EuroQol five-dimension five level VAS-score was a standardised questionnaire, used to assess the patient’s overall health status from 0-100. Higher score equal superior health [26].

*Pain:* Pain in the operated leg was measured using the four-point Verbal Rating Scale (VRS): 1–no pain, 2–slight pain, 3–moderate pain, 4–severe pain [27].

### Data collection and source

The older persons recorded the amount of IC received as the number of hours in a diary, Supplementary 1 [28, 29]. The data was collected by telephone interviews and home visits every two weeks from discharge to 12-week follow-up. The older persons were instructed to only record the new need for IC caused by the hip fracture and only the amount of time they received IC. For instance, if an informal caregiver provides help for bathing or grocery shopping as part of a longer visit, only the time the patient received care was to be recorded. Patients who did not fill in the diary were asked to estimate the hours of IC the previous week and to include both weeks; the estimate was multiplied by two. A Rehabilitation for Life trial physiotherapist collected demographic characteristics, type of surgery, NMS, CAS, Barthel-20, EuroQol five-dimension five-level VAS-score and VRS in the hospital on the day of discharge. Demographic characteristics and types of surgery were collected in the medical journals. NMS, CAS, Barthel-20, EuroQol five-dimension five-level VAS-score, and VRS were questionnaires the patients filled out in the hospital on the day of discharge. The physiotherapist read the questionnaires aloud for older persons with impaired vision.

### Sample size

The study size was determined from the number of participants in the Rehabilitation for Life trial [19].

### Statistical methods

Descriptive statistics for continuous variables were presented with medians and interquartile (IQR) due to non-normal distribution, while categorical variables were presented with frequencies and percentages. Group comparisons for continuous variables were performed using Wilcoxon’s rank sum test, and Pearson’s χ^2^ was used for categorical variables**.** The proportion of variance explained by variables differentiating recipients of IC from non-recipients and older persons’ high and low dependence at a 95% statistically significant level. The proportion of variance explained was assessed with McFadden pseudo-R2 and reported as the odds of receiving IC and high dependency, respectively. We used mono- and multivariate logistic regressions depending on the number of variables identified, differentiating persons receiving and not receiving IC and the high and low dependent persons at a statistically significant level. The statistically significant level was 95%. All statistical analyses were performed with StataCorp. 2019 (*Stata Statistical Software: Release 17*. College Station, TX: StataCorp LLC).

#### Drop out analysis

As not all older persons responded to the phone calls, an analysis between the older persons with complete and incomplete follow-up on discharge and demographic variables was completed.

#### Sub-analysis

Due to large IQR ranges of the median amount of IC, the median amount of IC from time of discharge to 12-week follow-up was used to create low and high dependence groups of older persons.

## Results

**Fig. 1:**
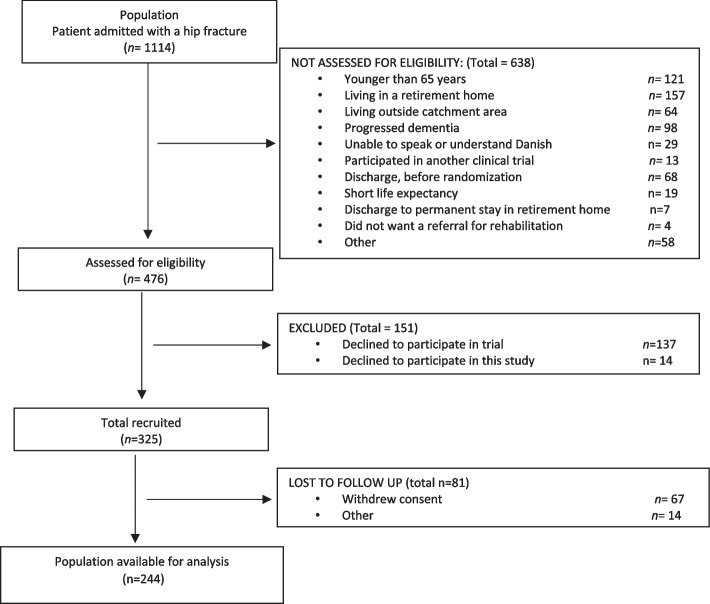
Flow chart of the inclusion process

From September 2020 to April 2023, 1.114 older persons were screened for study eligibility after hip fracture; of these 789 were excluded, leaving 244 older persons for inclusion (Fig. 1). The median age of the cohort was 78 (74-84) years; 66% were female, and 51% lived alone (Table 1).

**Table 1:** Demography percentage and hours of IC of the cohort and recipients and non-recipients of IC

Variables	No IC *n*= 25 (median IQR)	IC *n*=219 (median IQR)	Cohort *n*= 244 (median IQR)
Hours of informal care	0	32 (14-66)	27 (11-57)
Female *n* (%)	15 (60%)	146 (73%)	161 (66%)
Age	77 (70-83)	79 (74-84)	78 (74-84)
BMI	24 (21-28)	24 (21-28)	24 (21-28)
Living alone *n* (%)	13 (52%)	111 (51%)	124 (51%)
ASA score ≤ 2 *n* (%)	11 (44%)	121 (55%)	132 (54%)
Operation type *n* (%)^a^			
Arthroplasty	7 (28%)	75 (34%)	82 (34%)
Sliding hip screw	11 (44%)	48 (22%)	59 (24%)
Intramedullary nail	7 (28%)	95 (44%)	102 (42%)
NMS score	2 (1-4)	2 (1-3)	2 (1-3)
CAS score	6 (4-6)	5 (4-6)	6 (4-6)
Barthel-20	15 (10-18)	15 (11-17)	15 (11-17)
Overall Health	50 (33-75)	60 (50-75)	60 (50-75)
Pain operated leg			
No pain	3 (12%)	28 (13%)	31 (13%)
Slight pain	6 (24%)	54 (24%)	60 (24%)
Moderate pain	9 (36%)	87 (40%)	96 (40%)
Severe pain	7 (28%)	50 (23%)	57 (23%)

*IQR *Interquartile range, *BMI *Body mass index, *ASA *American Society of Anaesthesiologist Physical Status Score, *NMS *New Mobility Score, *CAS *Cumulated Ambulation Score

^a^marked variables differentiated the groups at a 95% significant level

### Number and percentage of older persons receiving IC

Of the 244 included older persons, 219 (90%) received IC. The median number of hours per week of IC from time of discharge to 12-week follow-up was 32 (14-66). Except for type of surgical treatment (*p*=0.049), at the baseline variables included in this study, the older persons who received IC were similar to older persons who did not receive IC (Table 1).

### Biweekly change in frequency and amount of IC

The number of older persons receiving IC and the number of hours of IC were highest in the first two to four weeks after discharge and declined over time. However, after twelve weeks, a third of the older persons still received informal care (Table 2). Approximately five to ten per cent of the older persons did not report on IC at each biweekly follow-up, and excluding older persons with missing information increased the biweekly amount of IC; the change has been visualised in Supplementary 2.

**Table 2:** Number of recipients and hours of informal care at each time point for the population and recipients of IC

	Week 1-2 *n*=221	Week 3-4 *n*= 234	Week 5-6 *n*=226	Week 7-8 *n*= 232	Week 9-10 *n*=216	Week 11-12 *n*=235
Receiving IC *n* (%)	157 (71%)	151 (65 %)	117 (52%)	126 (54%)	87 (40%)	84 (36%)
Cohort hours of IC median (IQR)	8 (0-27)	4 (0-14)	1 (0-7)	2 (0-8)	0 (0-4)	0 (0-4)
Recipients hours of IC median (IQR)	14 (8-28)	10 (4-20)	7 (4-16)	7 (4-18)	6 (3-15)	7 (4-17)
Missing *n*	23	10	18	12	28	9

### Drop out analysis

Of the 244 older persons, 63 (26%) had incomplete follow-up (Table 3). The older persons with complete follow-up received a median amount of IC of 28 (13-62) hours whereas the older persons with incomplete follow-up received a median of 14 (3-67) hours. Compared to the older persons with complete follow-up, the older persons with incomplete follow-up were older (*p*=.030), more frequently lived alone (*p*=.006), had higher ASA score (*p*=.026), surgically treated using intramedullary nails (*p*=.010), had poorer gait function (*p*= .000), had poorer basic mobility (*p*=.000), had poorer ability to perform activities of daily living (*p*=.001) and had poorer overall health (*p*=.005).

**Table 3:** Drop-out analysis between the older persons with complete and incomplete follow-up on baseline with demographics

Discharge	Complete follow-up *n*=180 Median (IQR)	Incomplete follow-up=64 Median (IQR)
Hours of IC	28 (13-62)	14 (3-69)
Female	120 (67%)	41 (64%)
Age^a^	78 (73-83)	80 (76-85)
BMI	24 (21-27)	23 (21-26)
Living alone^a^	82 (46%)	42 (65%)
ASA^a^	105 (58%)	27 (42%)
Operation type *n* (%)^a^		
Arthroplasty	69 (38%)	13 (21%)
Sliding hip screw	45 (25%)	14 (22%)
Intramedullary nail	66 (37%)	36 (57%)
Gait (NMS)^a^	2 (2-4)	2 (1-3)
Basic mobility (CAS)^a^	6 (4-6)	4 (3-6)
Barthel-20^a^	15 (12-17)	13 (9-16)
Overall Health^a^	60 (50-80)	50 (45-70)
Pain operated leg		
No pain	22 (12%)	9 (14%)
Slight pain	48 (27%)	12 (19%)
Moderate pain	75 (41%)	22 (34%)
Severe pain	36 (20%)	21 (33%)

*IQR* Interquartile range, *BMI* Body mass index, *ASA* American Society of Anaesthesiologist Physical Status Score, *NMS* New Mobility Score, *CAS* Cumulated Ambulation Score

^a^marked variables differentiated the groups at a 95% significant level

### Sub analysis

#### High and low dependence on IC

Of the 244 older persons, 110 (45%) had high dependence on IC (≥32 hours of IC) (Table 4). Older persons with high dependency received a median of 66 (46-107) hours of IC per week, and older persons with low dependency received a median of 11 (2-20) hours of IC. The two groups differed significantly from each other: compared with older persons with low dependency, the older persons with high dependency more frequently lived with a partner (*p*=.000), were more often surgically treated using intramedullary nail (*p*=.001), had poorer basic mobility (*p*=.019) and perceived their ability to perform basic activities of daily living as poorer (*p*=.040).

**Table 4:** Sub-analysis of the older person’s high or low dependence on IC

Variable	<32 hours of IC *n*=134 median (IQR)	≥32 hours of IC *n*=110 median (IQR)
Hours of IC	11 (2-20)	66 (46-107)
Female	86 (64%)	75 (68%)
Age	78 (73-83)	79 (75-84)
BMI	24 (21-28)	24 (21-27)
Living alone^a^	84 (63%)	40 (36%)
ASA score ≤2	68 (51%)	64 (58%)
Operation type *n* (%)^a^		
Arthroplasty	51 (38%)	31 (28%)
Sliding hip screw	40 (30%)	19 (17%)
Intramedullary nail	42 (32%)	60 (55%)
Gait (NMS)	2 (1-4)	2 (1-3)
Basic mobility (CAS)^a^	6 (4-6)	5 (3-6)
Barthel-20^a^	15 (12-17)	14 (11-16)
Overall Health	60 (50-75)	60 (50-75)
Pain operated leg		
No pain	19 (14%)	12 (11%)
Slight pain	36 (27%)	24 (22%)
Moderate pain	48 (35%)	48 (44%)
Severe pain	31 (23%)	26 (23%)

*IQR* Interquartile range, *BMI* Body mass index, *ASA* American Society of Anaesthesiologist Physical Status Score, *NMS* New Mobility Score, *CAS* Cumulated Ambulation Score

^a^marked variables differentiated the groups at a 95% significant level

### Variance analysis

#### Receiving IC

Univariate regression analysis did not indicate that the type of surgery increased the odds of receiving IC, and the proportion of variance explained was 1% (OR 1.12, 95% CI 0.701-1.818, R2 .016). No other variables differentiated recipients from non-recipients at a statistically significant level.

#### High dependency of IC

The univariate regression demonstrated that the odds of high dependence on IC increased by 135% if the patient was surgically treated using intramedullary nails. The type of surgery explained 4% of the difference between the older person’s high or low dependence on IC (OR 2.35 95% CI 1.295-4.236 R2 .04). Living with a partner increased the risk of being high dependent on IC by 194% and explained 5% of the proportion of variance (OR 2.94 95% CI 1.742-4.959 R2 0.05). Neither basic mobility (OR 0.83 95% CI 0.688, 1.009 R2 0.01) nor the ability to perform ADL activities (OR 0.93 95% CI 0.875, 1.000 R2 0.01) differentiate older persons with high dependence and low dependence at a 95% significance level. The multivariate regression included type of surgery, living arrangement, CAS and Barthel-20 score and combined these four variables explained 10.4% of the proportion of variance between older persons high or low dependent on IC. A table of the variance analysis are available in Supplementary 3.

## Discussion

### Key result

In this study, IC was very common, with 90% of the participants receiving IC with a median amount of 32 hours of IC in the 12 first weeks after discharge. The frequency and number of hours of IC were highest during the first two to four weeks after discharge and gradually declined over time. Sub-analysis demonstrated that the older persons high dependent on IC (≥32 hours) comprised 45% of the cohort; they received a median number of 66 hours of IC and were generally characterized as having poorer health and physical function at discharge compared to the older persons in the low dependent group. The variables of type of surgery and living with a partner explained 10% of the variance between the persons with high and low dependence on IC. Approximately one in four of the older persons did not have complete follow-up, and the older persons with complete follow-up differed from those with incomplete follow-up in having better health and physical function at discharge.

### Interpretation

During data collection, we were aware that older persons can be struggling with several diseases. During the pilot test, we learned that many of them failed to fill or incompletely fill their diaries [1, 30–32]. To mitigate this, we collected data via telephone interviews every two weeks, and non-responders to the telephone call were contacted twice on two separate days before a missing data point was accepted (i.e., a total of four telephone calls were performed). As a result, three out of four had complete follow-up, and none of the older persons with incomplete follow-up missed more than three follow-ups. Hence, we believe that the frequency of older persons receiving IC in this study is accurate.

The older persons in this study received a median of 32 hours of IC after discharge after hip fracture. To the best of our knowledge IC after hip fracture has not been quantified in health care system comparable to the Scandinavian before and the studies that have been conducted in Scandinavia have been of other populations’ than older persons after hip fracture [15, 33–36]. A study from the Netherlands have found that informal caregiver delivered a 39.5 hours of IC per week the first six months after hip fracture [36]. This difference might be due to the Netherlands’ mixed solidarity healthcare system where family and friends have an obligation to deliver IC [7]. Another key difference was that this study asked explicitly for the new need for IC after hip fracture and only asked the older persons to indicate the time they received IC. Given the very limited evidence, we can only recommend more research within this field.

Regarding the number of hours of IC, the sub-analysis of the older persons with missing information demonstrated that the older persons with incomplete follow-up had a lower median number of hours of IC and that their demographic and discharge characteristics more closely resembled those of persons highly dependent on IC. Thus, if all participants had a complete follow-up, the median number of hours of IC would likely have been higher. Hence, we recommend that the median amount of IC estimates be interpreted as minimum estimates, considering the older person’s physical level of function, as our estimates will likely best fit the proportion of older persons who were physically better at discharge after hip fracture. This finding was in line with Mathiowertz et al. 1994 [37] who argued that non-responders were often the most functionally limited persons. Mathiowertz et al. 1994 [37] found that the patients who lost levels of physical function were more inclined to have caregivers responding on their behalf.

Surgical procedure with intramedullary nail was associated with receiving IC, being highly dependent on IC and having incomplete follow-up. To our knowledge, these associations have not been identified before. However, because surgical approaches are planned with consideration of fracture type and location, recommendations of one procedure over another are likely ill-advised. The sub-analysis exploring the proportion of variance explained by type of surgery and living arrangement indicate a statically significant association to high dependence of IC and explained 4% and 5%, of variance between groups respectively. In a general context this may not be a great deal of variance explained, however it may indicate that it is possible to identify those with high dependence at discharge and prioritise resources accordingly. This however is beyond the scope of this study.

Based on the result of this study, informal caregiving is very common, and in our opinion, it is a positive matter that family and friends of patients want to take of their loved ones. Nevertheless, studies have shown that providing informal care wears on the caregivers with associations of increased morbidity, social isolation, and reduced quality of life [38]. This is, of course, not ideal, as caregivers should not become sick or worn out due to providing care for a loved one. Hence, we may need to consider if more support or a larger formal service level is needed, for patients with a high dependence on IC.

## Strength and limitations

This study has several strengths. First, due to the study’s novelty, a pilot test was completed in advance to identify and overcome potential challenges to obtaining an unbiased measure of IC [28, 39]. The data collection procedure was developed and feasibility tested in an iterative process involving 12 older persons who were followed for 12 weeks after discharge after hip fracture. Another clear strength of this study is the use of diaries and telephone calls to reduce missing information and recall bias.

An important limitation was the assumption that the amount of IC during the week the phone call was completed was representative of the previous week. As IC decreased over time it was probable that the older person received more IC in week three than in week four. Hence this assumption has potentially reduced the amount of IC. Another limitation is the size of the study population. With 244 older persons included, we did not have sufficient power to detect small differences.

### Generalizability

Generally, the Scandinavian countries are considered fairly homogenous [40]. Thus, and with respect to local differences, the results of the present study are probably applicable to other Scandinavian countries, but not necessarily to other countries directly. An important consideration for the generalizability of this study is the sample size. We included community dwelling and cognitively unimpaired older person, hence presented results are mainly representative for the healthier part of the hip fracture population.

## Conclusion

This study demonstrates that even though family and friends of older persons after hip fractures are not bound to deliver IC, the vast majority choose to do so. This was especially the case the first two to four weeks after discharge, and twelve weeks after discharge, a third of the older persons still received IC. We believe that this study was the first to quantify the older person’s need for IC after hip fracture in Scandinavia. Hence, we highly recommend more research within this area and the inclusion of IC in future health economic evaluations involving older persons after hip fracture. Furthermore, we believe the findings in the study emphasize the need to consider the impact of prioritisation on informal caregivers, at least to older people’s high dependence on IC. However, this will require additional resources.

### Supplementary Information

Supplementary Material 1.

Supplementary Material 2.

Supplementary Material 3.

## Data Availability

Data can be made available on reasonable request to corresponding author.
