# Supplementary material for: Informal care after hip fracture: prospective cohort
Source: BMC Geriatr. 2024 May 17;24:436. doi: 10.1186/s12877-024-05040-y (PMC11100116; doi:10.1186/s12877-024-05040-y)
Supplement: Supplementary file 1 — Supplementary Material 1. [file 12877_2024_5040_MOESM1_ESM.docx]

Supplementary 1. Translated cost diary used as a memory guide for the older persons

| Tabel 1. Relatives | | |
| --- | --- | --- |
| Uge | What type of assistance did you recieve | Time spent |
| Week 1 |  | Hours: Minutes: |
| Week 2 |  | Hours: Minutes: |
| Week 3 |  | Hours: Minutes: |
| Week 4 |  | Hours: Minutes: |
| Week 5 |  | Hours: Minutes: |
| Week 6 |  | Hours: Minutes: |
| Week 7 |  | Hours: Minutes: |
| Week 8 |  | Hours: Minutes: |
| Week 9 |  | Hours: Minutes: |
| Week 10 |  | Hours: Minutes: |
| Week 11 |  | Hours: Minutes: |
| Week 12 |  | Hours: Minutes: |
