# Supplementary material for: Informal care after hip fracture: prospective cohort
Source: BMC Geriatr. 2024 May 17;24:436. doi: 10.1186/s12877-024-05040-y (PMC11100116; doi:10.1186/s12877-024-05040-y)
Supplement: Supplementary file 2 — Supplementary Material 2. [file 12877_2024_5040_MOESM2_ESM.docx]

Supplementary 2. The change in percentage of older persons receiving IC and the median amount of hours.

Week 1-2 week 3-4 week 5-6 week 7-8 week 9-10 week 11-12
