# Supplementary material for: Informal care after hip fracture: prospective cohort
Source: BMC Geriatr. 2024 May 17;24:436. doi: 10.1186/s12877-024-05040-y (PMC11100116; doi:10.1186/s12877-024-05040-y)
Supplement: Supplementary file 3 — Supplementary Material 3. [file 12877_2024_5040_MOESM3_ESM.docx]

Supplementary 3. Supplementary 3. The proportion of variance explained by variables differentiating recipients from non-recipients and high dependent persons from low dependent persons at a statistically significant level.

|  | Variable | Odds ratio | 95% CI-interval | R^2^ |
| --- | --- | --- | --- | --- |
| Recipient of IC | Type of surgery | 1.12 | 0.701, 1.818 | 0.010 |
| High dependence | Type of surgery*  Living with a partner*  CAS score  Barthel-20 score  Combined | 2.35  2.94  0.83  0.93  - | 1.295, 4.236  1.742, 4.959  0.688, 1.009  0.875, 1.000  - | 0.040  0.050  0.010  0.011  0.104 |
| Cumulated Ambulation Score - CAS  associations significant at a 95% confidence level are marked by * | | | | |
